# P-1193. Epidemiology of Respiratory Syncytial Virus in Young, Hospitalized Children in Jordan: A Prospective Viral Surveillance Study

**DOI:** 10.1093/ofid/ofae631.1377

**Published:** 2025-01-29

**Authors:** Justin Z Amarin, Haya Hayek, Olla Hamdan, Yasmeen Z Qwaider, Tala Khraise, Ahmad Khader, Qusai Odeh, Rami Salim, Hadeel Shalabi, Ahmad Alhajajra, Yousef Khader, Basim Al-Zoubi, Najwa Khuri-Bulos, Andrew J Spieker, Leigh M Howard, James Chappell, Natasha B Halasa

**Affiliations:** Vanderbilt University Medical Center, Nashville, Tennessee; Vanderbilt University Medical Center, Nashville, Tennessee; Vanderbilt University Medical Center, Nashville, Tennessee; Vanderbilt University Medical Center, Nashville, Tennessee; Vanderbilt University Medical Center, Nashville, Tennessee; Vanderbilt University Medical Center, Nashville, Tennessee; Vanderbilt University Medical Center, Nashville, Tennessee; Vanderbilt University Medical Center, Nashville, Tennessee; Vanderbilt University Medical Center, Nashville, Tennessee; Al Bashir Hospital, amman, 'Amman, Jordan; Jordan University of Science and Technology, Irbid, Irbid, Jordan; Vanderbilt University Medical Center, Nashville, Tennessee; University of Jordan, Amman, 'Amman, Jordan; Vanderbilt University Medical Center, Nashville, Tennessee; Vanderbilt University Medical Center, Nashville, Tennessee; Vanderbilt University Medical Center, Nashville, Tennessee; Vanderbilt University Medical Center, Nashville, Tennessee

## Abstract

**Background:**

Respiratory syncytial virus (RSV) is a leading cause of acute respiratory infections in young children worldwide. With the recent wave of regulatory approvals and authorizations for nirsevimab and maternal RSV vaccines across several countries, understanding the current burden and seasonality of RSV is crucial for implementing effective preventive strategies globally. We aimed to determine the burden, seasonality, and co-detections of RSV in hospitalized children < 2 years old in Amman, Jordan.

**Figure 1.**
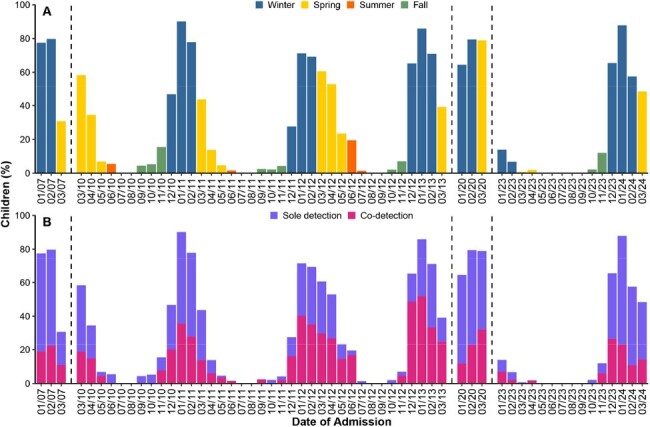
Monthly proportion of respiratory syncytial virus detections among children <2 years old hospitalized at Al-Bashir Hospital with fever or respiratory symptoms (N=5,922), described by (A) season and disaggregated by (B) co-detection status.

**Methods:**

We analyzed data from four prospective viral surveillance studies conducted at Al-Bashir Hospital collected during 2007, 2010–2013, 2020, and 2023–2024. We included children under 2 years old hospitalized with fever or respiratory symptoms who had a nasal or throat sample tested in the research laboratory for RSV and other common respiratory viruses using real-time polymerase chain reaction. Demographic and clinical data were collected through parental interviews and medical chart abstractions.

**Figure:**
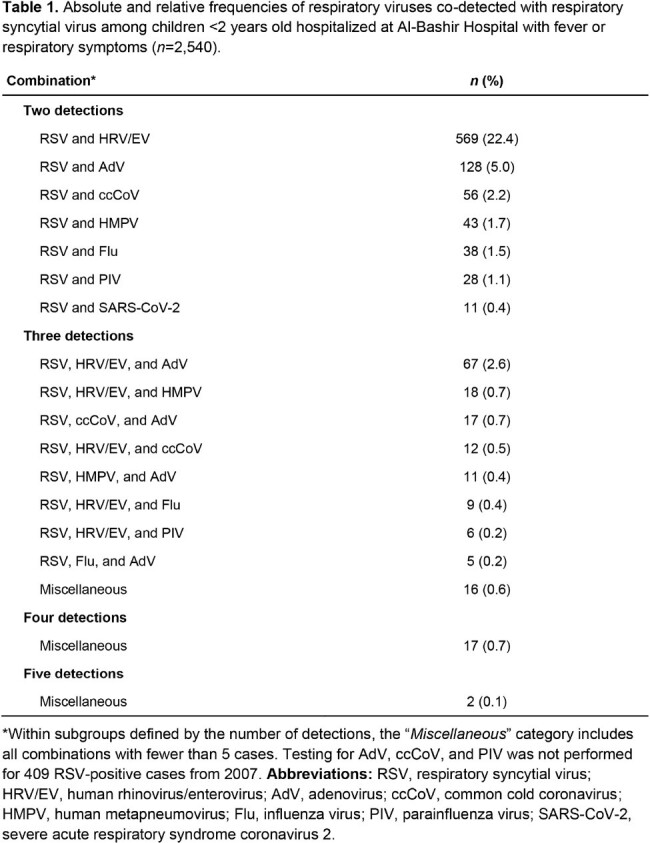

**Results:**

Among 5,922 children, 2,540 (42.9%) tested positive for RSV. The median age of children with RSV among this age-restricted cohort was 3.2 months (IQR, 1.6–6.8), and males represented 57.6% (*n*=1,463). RSV circulation showed seasonal peaks during winter months (**Figure 1**), with the highest proportions observed in January 2011 (90.1%), 2024 (87.9%), and 2013 (85.8%). The burden and seasonality of RSV during the 2023–2024 season appeared to normalize compared with the disrupted pattern observed in the preceding season. Co-detection of RSV with other respiratory viruses was observed in 41.5% of RSV-positive cases (**Table 1**), with the most common co-detected virus being human rhinovirus/enterovirus (22.4%), followed by adenovirus (5.0%).

**Conclusion:**

Our findings highlight the substantial burden of RSV among hospitalized young children in Amman, Jordan, and provide valuable insights into the current seasonality of RSV in a resource-limited setting. The normalizing circulation pattern has important implications for the timing and implementation of preventive strategies. The high proportion of RSV co-detections underscores the need for further research to elucidate the clinical significance of respiratory viral co-detection.

**Disclosures:**

**James Chappell, MD, PhD**, Merck: Grant/Research Support **Natasha B. Halasa, MD, MPH**, Merck: Grant/Research Support

